# Characterization of sediment bacterial communities in plain lakes with different trophic statuses

**DOI:** 10.1002/mbo3.503

**Published:** 2017-09-04

**Authors:** Wei Huang, Xing Chen, Xia Jiang, Binghui Zheng

**Affiliations:** ^1^ State Key Laboratory of Environmental Criteria and Risk Assessment Chinese Research Academy of Environmental Sciences Beijing China; ^2^ College of Environment and Planning Henan University Kaifeng China

**Keywords:** Bacterial community, lake, sediment, sequence, trophic status

## Abstract

Sediment microbial communities play an important role in lake trophic status. This study determined millions of Illumina reads (16S rRNA gene amplicons) to compare the bacterial communities in moderately eutrophic, lightly eutrophic, and moderately trophic regions using a technically consistent approach. The results indicated that the sediments from moderately eutrophic and trophic lake had the higher bacterial diversity than lightly eutrophic lake. *Proteobacteria* was the most abundant phylum (22.7%–86.2%) across samples from three regions. The sediments from moderately eutrophic region were enriched with *Chloroflexi* and *Nitrospirae*. *Alphaproteobacteria*,* Gammaproteobacteria*, and *Bacteroidetes* were enriched in the sediments from lightly eutrophic lake. The sediments from moderately trophic lake contained a high abundance of *Acidobacteria* and *Deltaproteobacteria* because of the low pH of the sediments in this lake. In moderately eutrophic region, *Nitrospira* held an absolute predominance, while *Lysobacter* and *Flavobacterium* were the most predominant genera in lightly eutrophic region. Temperature was the main factor influencing the bacterial community in the three lakes. The bacterial communities in the sediment samples obtained from moderately eutrophic lake were associated with nutrient concentration, whereas organic matter and total nitrogen contents mainly influenced the bacterial communities in sediments obtained from lightly eutrophic lake and moderately trophic lake, respectively.

## 1 INTRODUCTION

Lake sediment is one of the main media for nutrient transformation and migration and can act as either nutrient source or pool to the overlying water column; thus, sediments influence the nutrient contents of lakes. Microbial communities play an important role in nutrient cycling in aquatic ecosystems (Cotner & Biddanda, 2002; Jurgens et al., 2000; Ligi et al., 2014). Biogeochemical cycles, especially in sediments, are greatly influenced by microbial communities (Fang et al., 2015; Liu & Liu, 2013; Song, Li, Du, Wang, & Ding, 2012), and changes in the composition of sediment microbial communities can significantly impact the biogeochemical environment of sediments (Haukka et al., 2006; Van der Gucht et al., 2001). Trophic status is the structural and functional quality of water bodies; the trophic status of various aquatic ecosystems is assessed using different indices, such as biological communities and physical, chemical, morphological, and hydrological characteristics (Heiskanen, van de Bund, Cardoso, & Noges, 2004; Molozzi, Feio, Salas, Marques, & Callisto, 2012). The trophic status of aquatic ecosystems was greatly influenced by biological quality elements (Bere & Tundisi, 2010; Kelly et al., 2008; Teixeira et al., 2009). As one of the most critical factors influencing biological quality (Bending, Putland, & Rayns, 2000), microbial community has a significant relationship with trophic status (Duarte, Pascoal, Garabetian, Cassio, & Charcosset, 2009; Kostrzewska‐Szlakowska, 2005). In addition, sediment is a main component of lakes and contains complex microbial communities. Therefore, microbial communities in lake sediments can greatly influence the trophic status of lakes (Dai et al., 2016; Vezzulli & Fabiano, 2006).

Shallow fresh water lakes with different trophic statuses are commonly found in plains. They display different trophic statuses possibly because of various factors, such as amount of aquatic plants in lake (Ginn, 2011), exogenous pollution around the lake (Bertahas, Dimitriou, Karaouzas, Laschou, & Zacharias, 2006), and phytoplankton biomass (Phillips et al., 2013). As the trophic status of lakes changes, bacterial communities in sediments also change. In addition, the composition of bacterial communities can cause variations in nutrient contents of lakes (Molina et al., 2016; Phan et al., 2016). Therefore, trophic status and bacterial community are closely related, and understanding the differences in bacterial community structure of sediments in lake with different trophic statuses is crucial. This knowledge may improve the management and treatment of eutrophic lakes and the prevention of contamination of clean lakes.

In Eastern Plain of China, a large number of shallow fresh water lakes with different trophic statuses exist, and the sediments in these lakes greatly influence the trophic status of these lakes (Berglund, Larsson, Ewald, & Okla, 2001; Trolle, Hamilton, & Pilditch, 2010). Moreover, sediments in lake display higher biomass and microbe taxon richness than the water bodies (Huang & Jiang, 2016), and bacteria in sediments contribute to the decomposition of organic or inorganic compounds, promoting nutrient recycling (Liu et al., 2009; Nealson, 1997); as a result, nutrient contents in lake change, affecting the trophic status of lakes. Gonghu Bay is located in the northeast part of Taihu Lake (T), which is the third largest freshwater lake in China and located in the Eastern Plain of China. Gonghu Bay is an algae‐dominated region in Taihu Lake with an average depth of 2.02 m and an area of 147 km^2^. Nutrient contents have increased since 2005, and a massive algal bloom has occurred in Gonghu Bay. In 2007, the frequency of algal bloom increased greatly, and the algal bloom affected the security of drinking water in Taihu Lake Basin (Chen et al., 2010). The trophic state index (TSI) (Azevêdo et al., 2015) of Gonghu Bay has recently reached 64.8, and this area is classified as a moderately eutrophic region (Hou et al., 2013). Huangda Lake (HD), which is a shallow fresh water lake, is also located in the Eastern Plain of China. The area of Huangda Lake is approximately 269 km^2^, and its average depth is 3.64 m. Being a moderately trophic lake, Huangda Lake is a clean lake and its recent TSI value is approximately 45.4. East Dongting Lake (DT) is the largest region (1,328 km^2^) of Dongting Lake, which is the second largest freshwater lake in China. The East Dongting Lake is 2.1–7.3 m deep, and a large number of shallow areas exist in this region. With the increased anthropogenic activity, the trophic status of East Dongting Lake gradually increased in recent years, and its TSI reached up to 52.6 (in lightly eutrophic region).

This study investigated the bacterial communities in sediments obtained from the three lakes with different trophic statuses (moderately eutrophic, lightly eutrophic, and moderately trophic) using Illumina Miseq high‐throughput sequencing technology. A total of 5.1 million tags of the sediment samples were determined. This work will provide new insights into the nutrient control in lakes.

## 2 EXPERIMENTAL PROCEDURES

### 2.1 Sediment sampling

**Figure 1:**
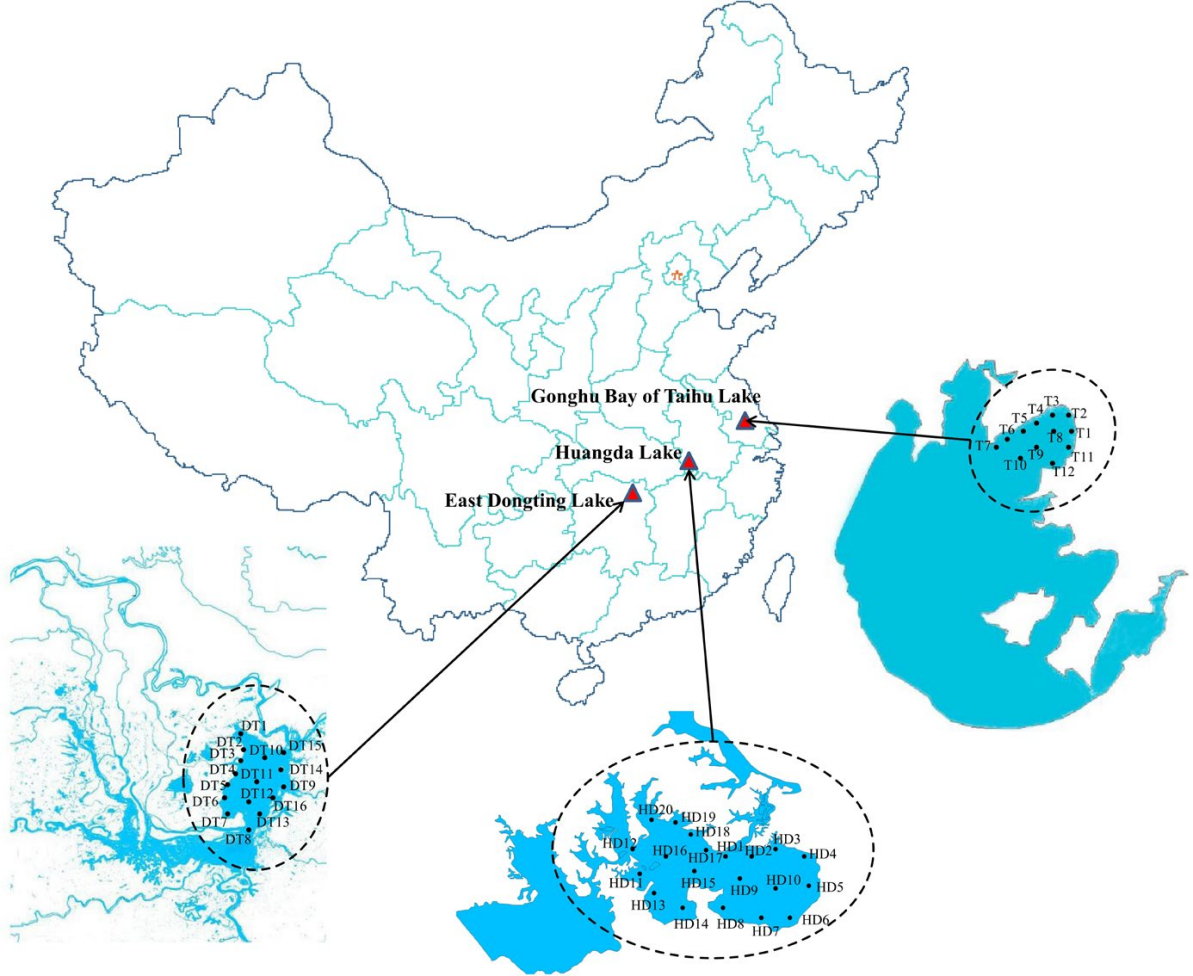
Sampling sites and location of the sampling lakes in China

A total of 48 samples were collected from Gonghu Bay of Taihu Lake (T, 12 samples), East Dongting Lake (DT, 16 samples), and Huangda Lake (HD, 20 samples) in August 2016. Figure**** 1 shows the sampling sites in three regions and the location of these lakes. The composite surface (10 cm) sediment samples (each *N = 5*) were collected in August 2016, and these samples were assigned as DT, HD, and T, respectively. The weight of the samples collected was sufficient for DNA extraction and for analysis of physicochemical parameters. After sampling, the sediment samples were placed in sealed plastic bags, stored in a portable ice box, transferred into the laboratory within 24 hr, and stored at −80°C before analysis.

### 2.2 Analysis of physicochemical parameters

The temperature of overlying water (Temp.), oxidation reduction potential (ORP) in sediments, and depth were measured in situ using a portable multi‐parameter water quality analyzer (ProPlus, YSI, USA). Total nitrogen (TN) and total phosphorus (TP) in sediments were measured using standardized methods and tests (Institute of Soil Science, Chinese Academy of Science, 1978; Ruban et al., 2001). Organic matter (OM) content in sediments was calculated according to the loss on ignition to constant mass (4 hr) at 550°C (Huang et al., 2015). The pH of the sediment was measured in a 1:2.5 (w/v) mixture of sediment and deionized water (Liao et al., 2014). Table**** 1 shows the main properties of the sediment samples.

**Table 1:** Physicochemical parameters in sediment and water from the three lakes with different trophic status

	T		DT		HD	
Parameter	Mean	Range	Mean	Range	Mean	Range
Depth (m)	2.01	1.42–3.11	5.28	0.31–7.35	3.64	2.16–5.27
Temp. (°C)	32.5	31.5–33.3	29.1	27.4–30.7	34.0	32.9–34.6
OM (%)^a^	4.55	3.92–6.37	9.21	7.21–11.02	7.51	5.06–10.25
ORP (mv)	35.7	−5.4–96.4	23.8	−21.3–123.6	49.1	−35.2–171.6
TN (mg kg^−1^)^a^	1708.1	1391.2–2170.4	912.6	611.9–1423.2	1969.5	1157.3–3333.7
TP (mg kg^−1^)^a^	793.1	631.9–891.6	823.6	602.3–1003.7	571.9	386.1–797.1
pH^a^	7.85	7.16–8.31	7.15	7.11–7.46	6.21	5.47–7.27

Data of T, DT, and HD are obtained from 12, 16, and 20 sites, respectively.

a Significant differences exist among the three lakes (*p *<* *.05).

### 2.3 Extraction of genomic DNA

Total genomic DNA was extracted from 250 mg samples using a PowerSoil DNA Isolation Kit (Mobio Laboratories Inc., San Diego, CA, USA) according to the manufacturer's protocol. DNA concentration and quality were determined using a NanoDrop Spectrophotometer. DNA was diluted to 10 ngμl^−1^ using sterile ultrapure water and stored at −80°C for further analysis.

### 2.4 PCR amplification of 16S rRNA genes and sequencing

The barcode 515F (5ʹ‐GTGCCAGCMGCCGCGGTAA‐3ʹ) and 926R (5ʹ‐CCGTCAATTCMTTTGAGTTT‐3ʹ) primers were used to amplify the V4–V5 regions of the bacterial 16S rRNA (Liu, DeSantis, Andersen, & Knight, 2008; Wang & Qian, 2009; Xiong et al., 2014). The PCR mixture (25 μl) contained 1 ×  PCR buffer, 1.5 mmol/L MgCl_2_, 0.4 μmol/L each of deoxynucleoside triphosphate, 1.0 μmol/L of each primer, 0.5 U of TaKaRa *Ex Taq*, and 10 ng of template DNA. The PCR amplification program is as follows: initial denaturation at 94°C for 1 min followed by 30 cycles (denaturation at 94°C for 20 s, annealing at 56°C for 30 s, and elongation at 72°C for 45 s) and a final extension at 72°C for 5 min. Three replicates of PCR reactions for each sample were combined. A similar volume of 1 ×  loading buffer (containing SYB green) was mixed with PCR products and then subjected under electrophoresis on 2% agarose gel for detection. Samples showing bright main strip of 300–500 bp were chosen for further experiments. PCR products were purified using an OMEGA Gel Extraction Kit (Omega Bio‐Tek, USA), and equal molar amounts of the products from different samples were pooled. Sequencing libraries were generated using TruSeq DNA PCR‐Free Sample Prep Kit according to the manufacturer's recommendations, and index codes were added. Library quality was assessed on a Qubit@2.0 Fluorometer (Thermo Scientific) and Agilent Bioanalyzer 2100 system. Finally, the library was subjected under paired‐end sequencing (2 × 250 bp) on an Illumina Miseq apparatus at Rhonin Biosciences Co., Ltd.

### 2.5 Statistical analysis

After sequencing, Usearch (Edgar, 2010; Edgar, Haas, Clemente, Quince, & Knight, 2011) and QIIME pipeline were used for analysis (Caporaso et al., 2010). Paired‐end reads from the original DNA fragments were merged using FLASH (Magoc & Salzberg, 2011). The sequences were assigned to each sample according to the unique barcode. In this study, we adopted relatively stringent quality controls. We first filtered low‐quality reads (length is < 200 bp, more than two ambiguous base “N,” or average base quality score is < 30) and truncated sequences, wherein quality scores decay (score < 11). After finding the duplicated sequences, we discarded all singletons, which are possibly bad amplicons that lead to overestimation of diversity. Sequences were clustered into operational taxonomic units (OTUs) at 97% identity threshold using UPARSE algorithms (Edgar, 2013). We selected representative sequences and removed potential chimeras using UCHIME algorithm (Edgar et al., 2011). The clean sequences of each sediment sample ranged from 17,770 to 25,9634.

Principal coordinate analysis (PCoA) was employed to explore and visualize the similarities between sediment samples obtained from lakes with different trophic statuses based on Bray–Curtis dissimilarity using package Ape (Paradis, Claude, & Strimmer, 2004). Distance‐based redundancy analysis (db‐RDA) was performed in R environment using the package Vegan. The Pearson correlation method was used to analyze the correlation analysis between genera and environmental variables, and p values were corrected by FDR (False Discovery Rate) method. Then the complete‐linkage clustering of OTU was used to obtain the clustering information which was present using heatmap. The functions Capscale, Anova.cca, and Vif.cca in R package Vegan were used to perform model construction, variables or axes significance permutation test, and variance inflation factor analysis. LEfSe (University of Auckland, Auckland, New Zealand) was used to identify the indicator bacterial groups specific to the sediment samples (Segata et al., 2011). In addition, independent T‐test and permutational multivariate analysis of variance based on Bray–Curtis dissimilarity matrix were used to identify the differences that exist among different groups.

### 2.6 Accession numbers

All of the sequencing data analyzed in this study can be downloaded from the NCBI's Sequence Read Archive using the accession numbers SRR5131114, SRR531118, and SRR5131121 for samples from Gonghu Bay of Taihu Lake, East Dongting Lake, and Huangda Lake, respectively.

## 3 RESULTS

### 3.1 Diversity indices of bacterial community

**Figure 2:**
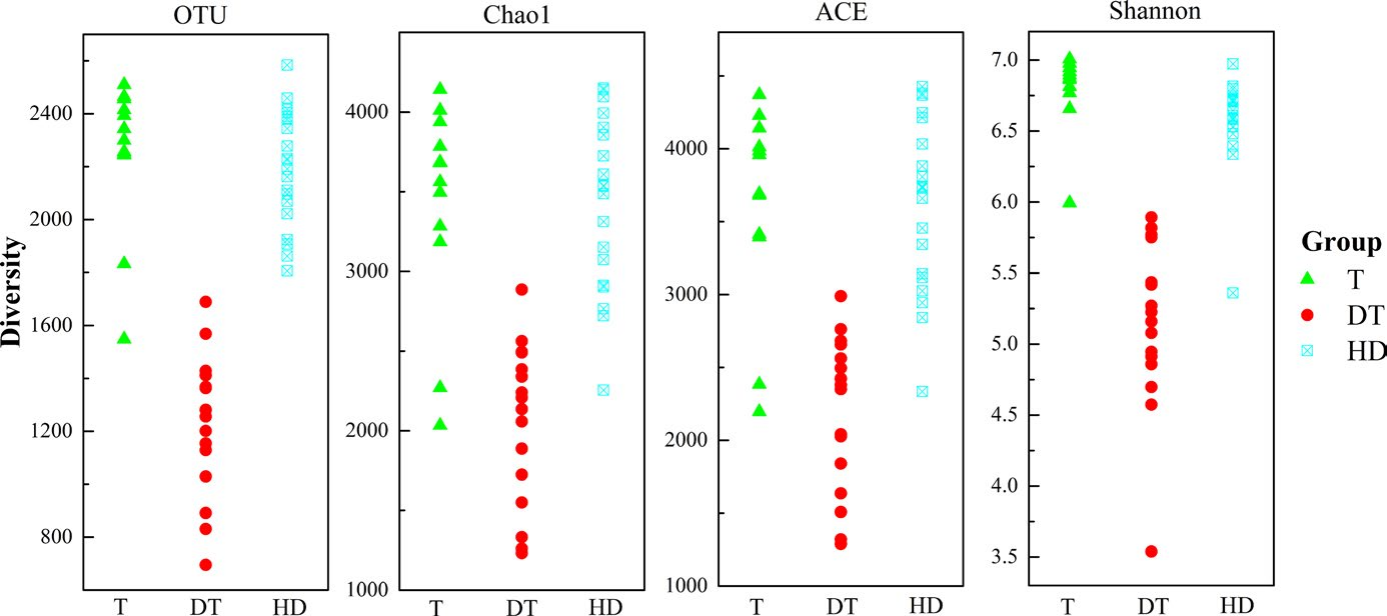
Diversity indices of 16S rRNA gene libraries with significant differences (*p *<* *.05)

A total of 48 sediment samples were obtained from moderately eutrophic, lightly eutrophic, and moderately trophic lakes. Usearch and QIIME pipeline were used to analyze the raw sequences. Relatively representative sequences were selected and potential chimeras were removed using UCHIME algorithm. Figure 2 shows the values of diversity (Chao1, ACE, and Shannon) and OTU in sediments obtained from the three lakes. After sequencing and quality control, the observed OTU in East Dongting Lake was 1,229 in average, which was lower than that in Gonghu Bay of Taihu Lake (2,251) and Huangda Lake (2,193). Chao1, ACE, and Shannon indices of the bacterial communities in sediments obtained from Gonghu Bay of Taihu Lake and Huangda Lake showed similar values. The value of Chao1 varied from 2023.4 to 4141.6 (2423.0 in average) and 2255.3 to 4194.9 (3430.7 in average) for Gonghu Bay of Taihu Lake and Huangda Lake, respectively. The value of Chao1 in East Dongting Lake was lower than that in the two other lakes and varied from 1232.5 to 2886.1 (2049.5 in average). Moreover, ACE of bacterial communities in sediments from Gonghu Bay of Taihu Lake and Huangda Lake were higher than that in sediments obtained from East Dongting Lake (*p *<* *.05). Shannon index of DT13 in East Dongting Lake showed the lowest value (3.54), and the other positions varied from 4.57 to 5.89 (5.15 in average), which was significantly lower than that in Gonghu Bay of Taihu Lake (6.80 in average) and Huangda Lake (6.57 in average) (*p *<* *.05).

### 3.2 Bacterial community composition in sediment samples

**Figure 3:**
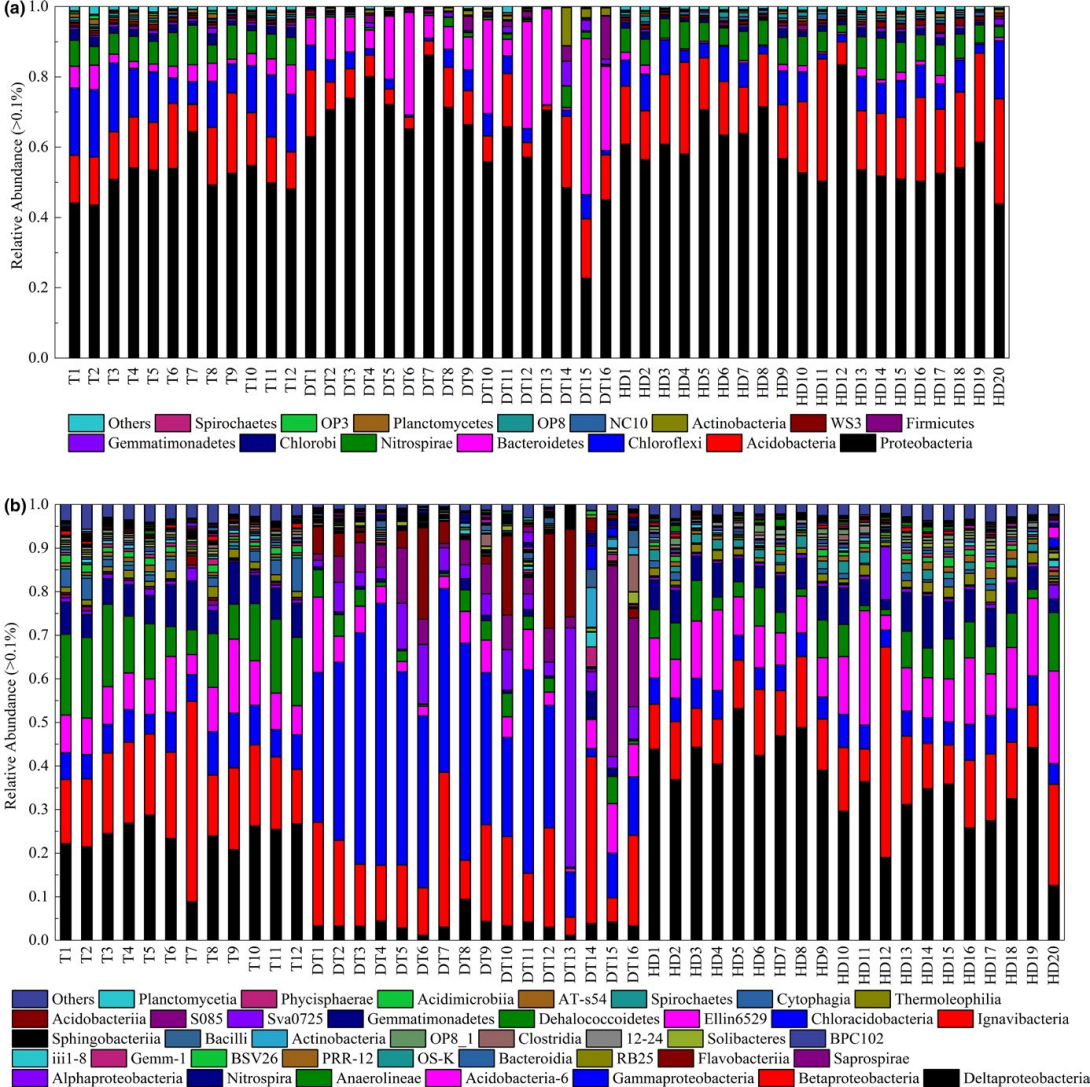
Abundances of different phyla (a) and classes (b) in 48 sediment samples based on pyrosequencing at 0.03 level (12, 20, and 16 samples from Gonghu Bay of Taihu Lake, Huangda Lake, and East Dongting Lake, respectively)

Among the filtered sequences, 56 phyla were determined in the sediment samples obtained from the three lakes. Figure 3 shows the dominant groups (relative abundance >0.1%) in each sample. *Proteobacteria* was the most abundant phylum (22.7%–86.2%) across samples, and the bacterial sequences were affiliated with *Acidobacteria* (12.9%–34.7%), *Chloroflexi* (2.5%–19.6%), *Bacteroidetes* (2.5%–44.4%), and *Nitrospirae* (1.3%–11.8%). Figure 3a shows the differences in bacterial communities at the phylum level between the sediment samples from three regions. The diversity of *Chloroflexi* and *Nitrospirae* was more abundant in the sediment samples obtained from Gonghu Bay of Taihu Lake and Huangda Lake than in the samples obtained from East Dongting Lake, and significant differences (*p *<* *.01) were observed among the samples obtained from these lakes. *Firmicutes* was more abundant in the sediments obtained from East Dongting Lake (e.g., 4.0% in DT9 and 12.3% in DT16) than in the sediments obtained from Gonghu Bay of Taihu Lake and Huangda Lake. *Gemmatimonadetes*,* Actinobacteria*,* Sporochaetes*,* Planctomycetes*,* OP8*,* NC10*, and *WS3* were the other phyla which were universally detected in the sediment samples from three regions.

Figure**** 3b shows the changes in bacterial community composition at the class level. *Deltaproteobacteria*,* Betaproteobacteria*,* Gammaproteobacteria*,* Alphaproteobacteria*,* Anaerolineae*, and *Nitrospira* predominated in the three lakes. The sediment samples obtained from Gonghu Bay of Taihu Lake and Huangda Lake displayed higher abundance of *Deltaproteobacteria* (23.2% and 36.3%, respectively) than the samples obtained from East Dongting Lake (3.6%); *Nitrospira* is also more abundant in the Gonghu Bay of Taihu Lake and Huangda Lake (7.5% and 6.9%, respectively) than in East Dongting Lake (1.2%). In addition, *Gammaproteobacteria* and *Flavobacteria* were more abundant in the sediment samples obtained from East Dongting Lake than in the samples obtained from Gonghu Bay of Taihu Lake and Huangda Lake (*p *<* *.01). *Saprospirae*,* Bacteroidia*,* Solibacteres*,* Closstridia*,* Actinobacteria*,* Bacilli*,* Dehalococcoidetes*,* Gemmatimonadetes*, etc. were the other classes which could be detected in the sediment samples from three regions.

**Figure 4:**
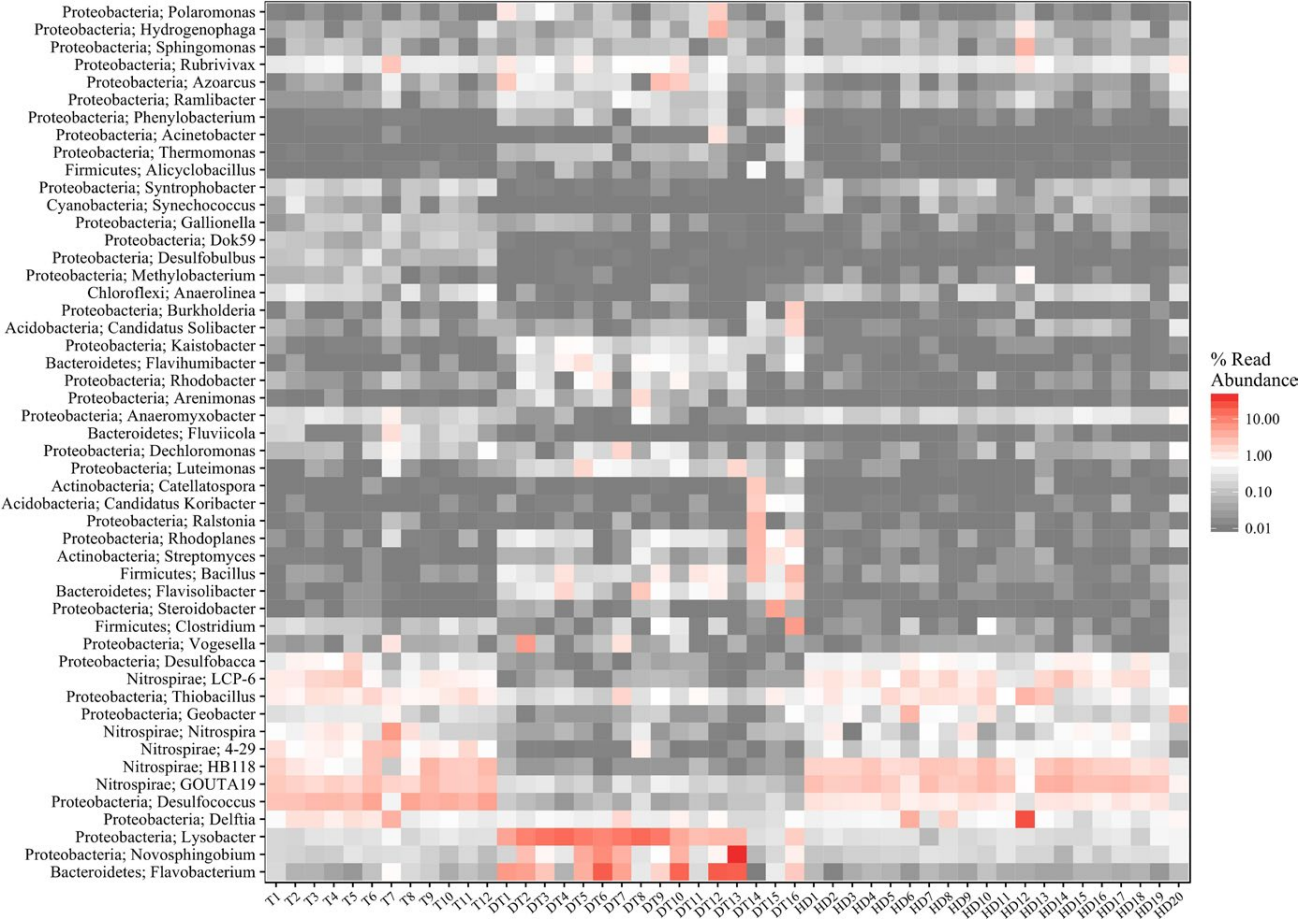
Heatmap analysis of bacterial community at genus level

The taxonomic composition at the genus level of the bacterial communities in sediment samples from three regions is shown in Figure 4 **.** *Lysobacter* and *Flavobacterium* were the most predominant genera in most of sediment samples from East Dongting Lake (with relative abundance, >7%). The relative abundance of *Nitrospira* in sediment samples from Gonghu Bay was higher than other two regions (with relative abundance, >5%). *HB118*,* GOUTA19*, and *Delftia* in the sediment samples from Huangda Lake had higher relative abundance than other two regions. In addition, other dominant genera with appreciable relative abundance (average values above 0.1%) in the sediment sample from the three regions included *Desulfococcus, Geobacter, Anaerolinea, Methylobacterium, Desulfobulbus, Hydrogenophaga, Sphingomonas, Polaromonas, Rubrivivax*, etc.

### 3.3 Principal coordinate analysis

**Figure 5:**
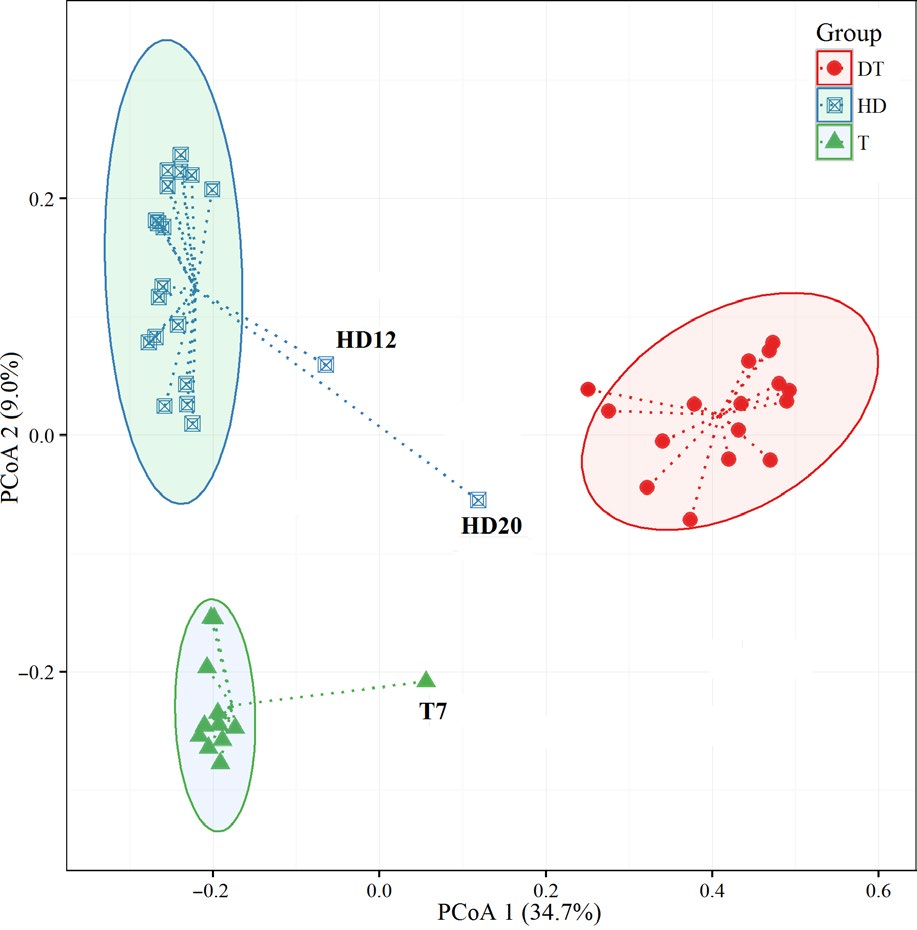
Ordination plot showing the grouping of the sediment samples from the lakes with different trophic statuses according to their bacterial community structure; the principle coordination analysis was based on Bray–Curtis distance matrix

PCoA revealed the differences in microbial community patterns among sediment samples obtained from the three lakes. Figure 5 shows the grouping of the sediment samples according to their bacterial community structure. Two PCoAs explained 43.7% of the total variation in the microbial community structure. Bacterial communities were clearly clustered in the ordination plot according to sediment samples (Figure**** 5), with the samples from Gonghu Bay of Taihu Lake (T), East Dongting Lake (DT), and Huangda Lake (HD) separated from each other. In addition, the samples from HD20 and HD12 were separated from other samples obtained from Huangda Lake, and the sediment samples of T7 showed similarity with the other samples from Gonghu Bay of Taihu Lake. Independent T‐test indicated that the bacterial communities in sediment samples from the three lakes significantly differed from one another (*p *<* *.05).

### 3.4 Distance‐based redundancy analysis (db‐RDA)

**Figure 6:**
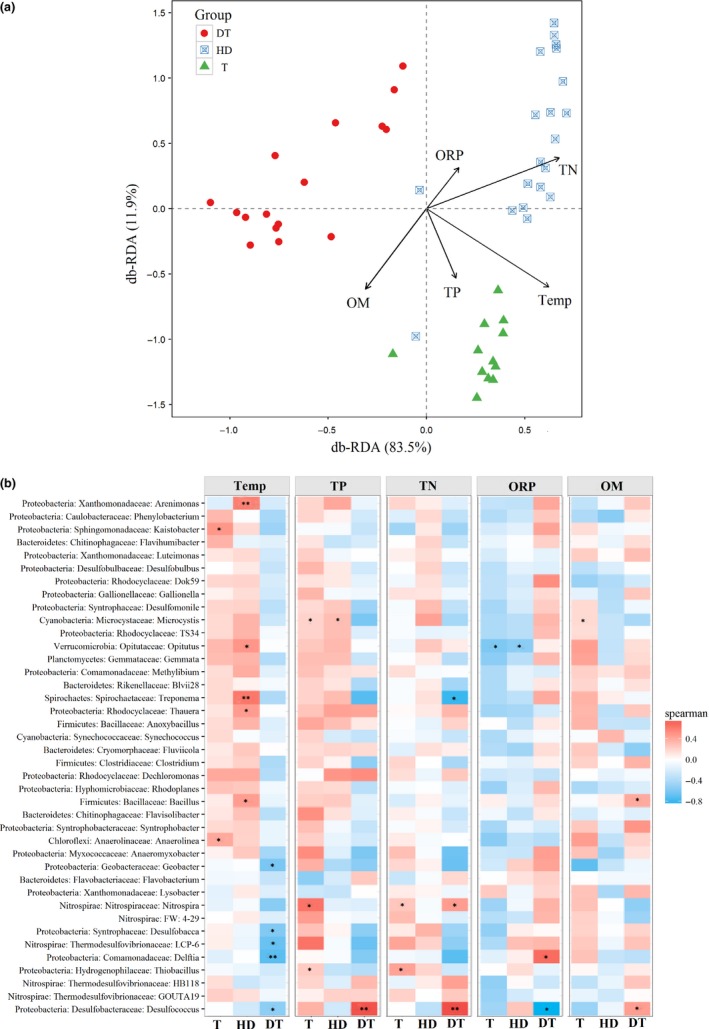
Relationship between bacterial communities and physiochemical parameters in three lakes with different trophic statuses. (a) Structure of bacterial community composition constrained by physiochemical parameters. (b) Correlation analysis between genera and physiochemical parameter. * *p *<* *.05, **p <* *.01

db‐RDA was used to partition the variation in beta‐diversity into fractions based on environmental variables (physiochemical parameters). Figure**** 6a shows the relationship between bacterial community composition and physiochemical parameters. The db‐RDA results suggest that the bacterial community composition of the sediment samples changed with trophic status. The bacterial communities in East Dongting Lake were associated with OM content; by contrast, those in the Huangda Lake were mainly associated with TN concentration, whereas some communities were associated with ORP. In Gonghu Bay of Taihu Lake, multiple physiochemical parameters, such as TN, TP, OM, and temperature, could affect the bacterial community in sediments, resulting in the differences in these communities from those in the two other lakes.

Figure 6b shows the correlation analysis between genera and physicochemical parameters based on db‐RDA. Temperature is the main influencing factor in bacterial community at the genus level in the three lakes. The genus *Microcystis* (phylum *Cyanobacteria*) was positively correlated with TP concentration (*p *<* *.05) in sediments obtained from Gonghu Bay of Taihu Lake and Huangda Lake, as well as correlated with OM content (*p *<* *.05) in sediments obtained from Gonghu Bay of Taihu Lake. Moreover, the db‐RDA results show that in Gonghu Bay of Taihu Lake, *Nitrospira* and *Thiobacillus* were associated with TN concentration (*p *<* *.05). In the sediments of East Dongting Lake, *Desulfococcus* (phylum *Proteobacteria*) was positively correlated with TP, TN (*p *<* *.01), and OM content (*p *<* *.05). In addition, *Bacillus*,* Treponema*, and *Opitutus* were correlated with OM, temperature, and ORP, respectively.

### 3.5 LEfSe analysis based on community abundance

**Figure 7:**
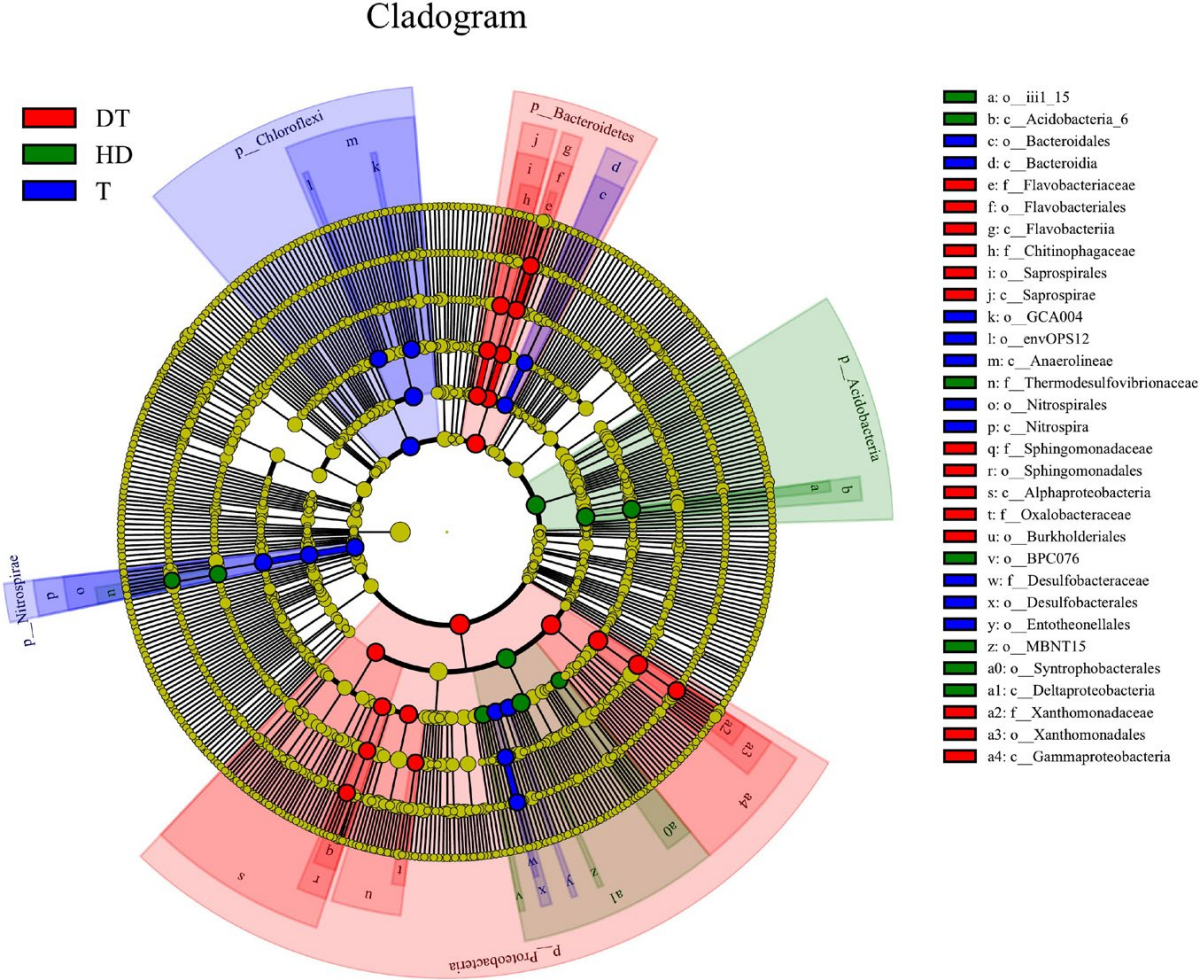
Cladogram showing the phylogenetic distribution of microbial lineages associated with the sediments in lakes with different trophic statuses; lineages with LDA values of 4.0 or higher as determined by LEfSe are shown. Differences are represented by the color of the most abundant class (red indicating East Dongting Lake, green, Huangda Lake, and blue, Gonghu Bay of Taihu Lake; yellow represents insignificant difference). The diameter of each circle is proportional to a taxon's abundance. Circles from inner region to outer region represent the phylogenetic levels from domain to genus

**Figure 8:**
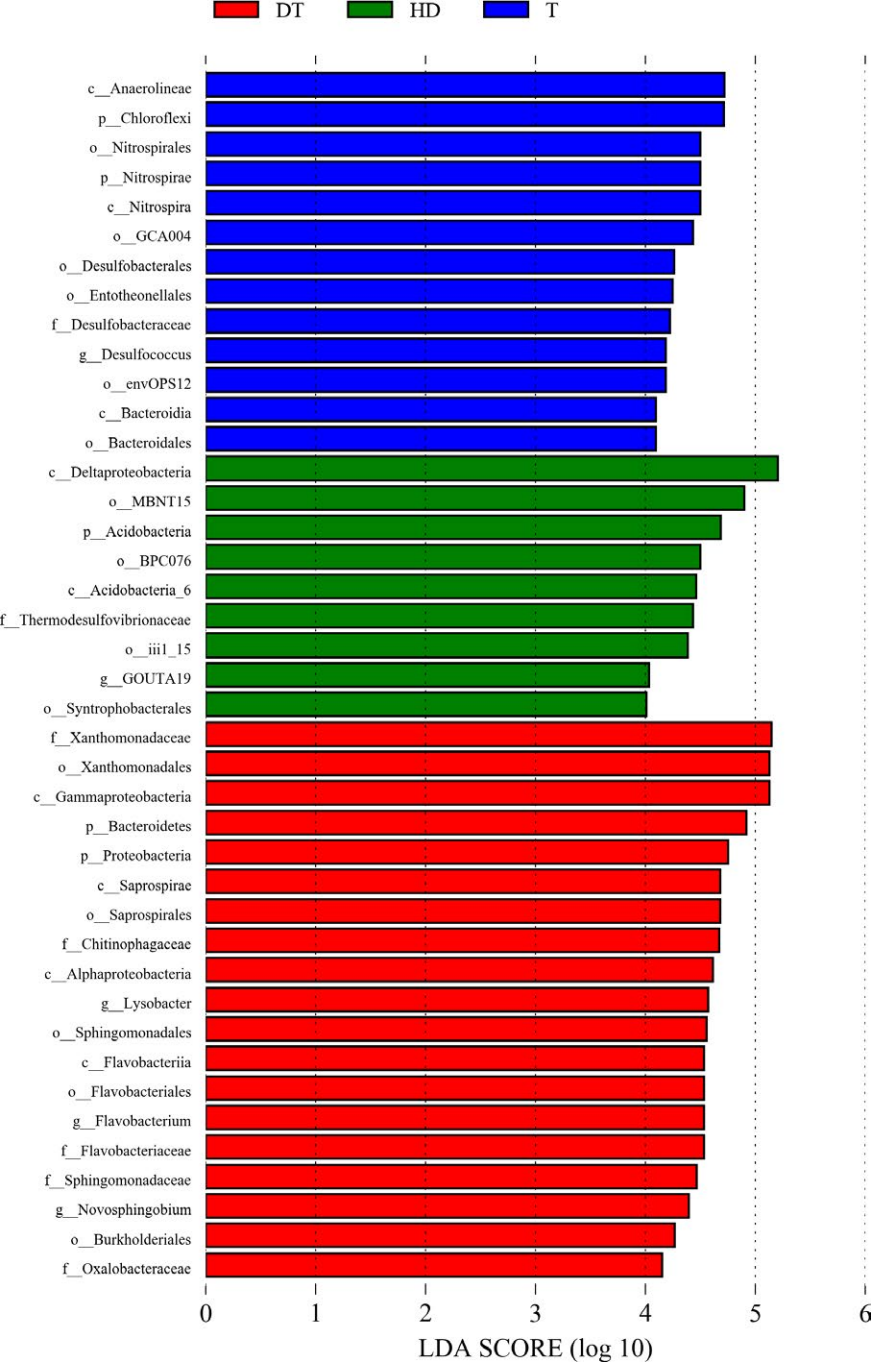
Indicator microbial groups in the three group of sediment samples with LDA values higher than 4.0

Figure 7 shows that the bacterial lineages enriched in sediment samples from Gonghu Bay of Taihu Lake were *Chloroflexi*,* Nitrospirae* (from phylum to order), *Bacteroidia* (the class and order of *Bacteroidales*), *Desulfobacterales* (the class and order of *Desulfobacteraceae*), and *Entotheonellales* (within *Deltaproteobacteria*). Within these groups, six fine lineages showed an LDA value of 4 or higher; these lineages are *Nitrospirales*,* GCA004*,* Entotheonellales*,* Desulfobacteraceae*,* envOPS12*, and *Bacteroidales* (Figure 8).

Phylum *Proteobacteria* (Figures 7 and 8), particularly *Alphaproteobacteria*, one order in *Gammaproteobacteria* (*Xanthomonadales*), and two classes in *Bacteroidetes* (*Flavobacteriia* and *Saprospirae*) were enriched in the sediments obtained from East Dongting Lake. At the fine taxonomy levels, *Xanthomonadaceae*,* Chitinophagaceae*,* Flavobacteriaceae*,* Sphingomonadaceae*, and *Oxalobacteraceae* found in the sediment samples from East Dongting Lake showed LDA values of higher than 4 **(**Figure 8).

In the sediment samples from Huangda Lake, *Thermodesulfovibrionaceae* (a family within *Nitrospirae*), *Acidobacteria*, and *Deltaproteobacteria* (the class and orders of *Syntrophobacterales*,* MBNT15*, and *BPC076*) were enriched in bacterial community (Figure**** 7). In the sediment samples from Huangda Lake, *Thermodesulfovibrionaceae* and three groups of *Deltaproteobacteria* showed LDA values of higher than 4 (Figure 8).

## 4 DISCUSSION

*Proteobacteria* is nearly the most abundant phylum in the three districts. *Proteobacteria* is commonly the most abundant phylum in sediment or soil and that it strongly plays a role in degradation and metabolism in lake sediments (Bai et al., 2012; Chaudhry, Rehman, Mishra, Chauhan, & Nautiyal, 2012). In the Taihu Lake Basin, amounts of exogenous pollutants entered into Taihu Lake, especially in Gonghu Bay, making the latter moderately eutrophic. The sedimentation of organics occurred, and *Chloroflexi*, which is highly abundant, participated in organic degradation (Daniel, Pozzi, Foresti, & Chinalia, 2009). Therefore, the OM content of sediments was relatively lower in Gonghu Bay of Taihu Lake than in two other lakes (Table**** 1). In lightly eutrophic lake (East Dongting Lake), *Firmicutes* predominated in some sediment samples, such as DT9 and DT16. Every August, the water level in Dongting Lake is low, and dry‐rewet cycles occur, especially in East Dongting Lake. Therefore, some sampling sites show high abundance of *Firmicutes*, which can produce spores that resist dehydration and extreme environmental conditions (Huang & Jiang, 2016).

Studies have indicated that *Gammaproteobacteria* existed in organic‐rich sediments, such as in the sediments in areas contaminated with agricultural pollution or organic sediments (Beardsley, Moss, Malfatti, & Azam, 2011; Bissett, Bowman, & Burke, 2006; Kunihiro et al., 2011). Anthropogenic activities and agricultural pollution around the basin of East Dongting Lake have recently worsened, and amounts of organic pollutants have entered into East Dongting Lake, worsening the trophic status of the lake. The organic content of sediments increased due to the sedimentation of exogenous organic pollutants. As a result, *Gammaproteobacteria* in sediment samples is more abundant in East Dongting Lake than in the two other lakes. In addition, *Flavobacteria* played a significant role in biopolymer degradation in sedimentary organic matter (Bauer et al., 2006; Bissett, Bowman, & Burke, 2008; Gomez‐Pereira et al., 2012) and was more highly abundant in East Dongting Lake than in the two other lakes. *Deltaproteobacteria* and *Nitrospira* predominated in the sediment samples from Gonghu Bay of Taihu Lake, which is an algae‐dominated and moderately eutrophic area and where massive algal bloom occurs every August. In Gonghu Bay of Taihu Lake, *Deltaproteobacteria* and *Nitrospira* are possibly the important classes for nutrient release in sediments, and the amount of these two classes can serves as potential early warning for the occurrence of algal bloom. The distribution of bacterial community in sediments obtained from Huangda Lake at the class level is similar to that in the sediments obtained from Gonghu Bay of Taihu Lake, indicating that although Huangda Lake is a moderately trophic lake, the nutrient in sediments can be potentially released. Therefore, being a clean lake, the sediments of Huangda Lake must be treated to retain the better water quality.

Sediments from Huangda Lake, which is moderately trophic lake, showed a unique pattern of bacterial diversity (phylum of *Acidobacteria*) compared with that in East Dongting Lake (lightly eutrophic lake) and Gonghu Bay of Taihu Lake (moderately eutrophic lake). In addition, the bacterial groups enriched in Huangda Lake were mainly limited to *Nitrospirae* and *Deltaproteobacteria* (Figures 3a and b). Huangda Lake is a freshwater and moderately trophic lake, and *Acidobacteria* are ubiquitous and abundant members of bacterial communities in the sediment of freshwater lake. Studies have suggested that abundance of *Acidobacteria* is significantly correlated with pH, and *Acidobacteria* prefers an environment with low pH of approximately 5.5 (Jones et al., 2009; Lauber, Hamady, Knight, & Fierer, 2009). The pH of sediments in Huangda Lake is lower than that in sediments of East Dongting Lake and Gonghu Bay of Taihu Lake (Table**** 1), possibly resulting in higher abundance of *Acidobacteria* in Huangda Lake than in the two other lakes (Figure 3a). Furthermore, LEfSe analysis showed that subdivisions 6 (*Acidobacteria_6*) are enriched in the sediments from Huangda Lake (Figure 7), indicating that the distribution of *Acidobacteria* subdivisions in lakes varies with trophic status (Orcutt, Sylvan, Knab, & Edwards, 2011). The trophic status in East Dongting Lake has recently increased, and the sediments played a significant role in the trophic status of the lake. Several microbes (e.g., *Bacteroidetes*,* Firmicutes*,* Alphaproteobacteria*, and *Gammaproteobacteria*), which prefer eutrophic conditions, were enriched in sediments obtained from East Dongting Lake, and the bacterial community in the sediment possibly affected the trophic status of the lake. *Chloroflexi* and *Nitrospirae* (from phylum to order) were mainly enriched in the sediments obtained from Gonghu Bay of Taihu Lake (Figure 7). *Nitrospira* from *Nitrospirae* was the most abundant genus in the sediments obtained from Gonghu Bay of Taihu Lake, and nitrogen is possibly one of the main limiting factors of eutrophication instead of phosphorus in Taihu Lake; *Nitrospira* is a strong indicator of nitrogen cycle. *Chloroflexi* is a photoautotrophic microbe and has possibly participated in the degradation of organics, thereby influencing nutrient content, which is one of the main factors affecting trophic status. Therefore, *Nitrospirae* or *Chloroflexi* can potentially serve as warning for the occurrence of algal bloom.

The bacterial communities in sediment samples from Gonghu Bay were influenced by TP, TN, temperature, and OM. Studies have indicated that TP and temperature are the main factors influencing bacterial communities, and phosphorus is a significant limiting factor of eutrophication in Taihu Lake (Shao et al., 2011; Song et al., 2012; Tang et al., 2009). In addition, Gonghu Bay is an algae‐dominated area in Taihu Lake, and the concentration of nutrients, such as TN and TP, should stabilize the bacterial community in the sediments. Therefore, TP and temperature were the main environmental factors affecting the bacterial communities. Gonghu Bay of Taihu Lake, which is a moderately eutrophic lake, suffers from serious eutrophication every August, and the growth and metabolism of algae is significantly related to the sediments. The genus *Microcystis* (phylum *Cyanobacteria*) is positively related to TP concentration, demonstrating that P remains one of the most significant limiting factors of eutrophication in Gonghu Bay of Taihu Lake, and the sediments of the algae‐dominated area is dominated by *Microcystis* (Shao et al., 2011; Wu, Chen, Xu, Liu, & Hahn, 2007). In addition, *Microcystis* is positively correlated with TP concentration in sediments from Huangda Lake. Huangda Lake is a clean lake and has a moderately trophic status with TSI of 45.4, and the average TP concentration in sediments is 571.9 mg kg^−1^ (Table**** 1). However, the TP concentration in sediments or water has recently increased due to fish farming and mining of phosphate near Huangda Lake; these anthropogenic activities possibly promote the activity of *Microcystis* (phylum *Cyanobacteria*) community, increasing the risk of eutrophication.

Although P is a limiting factor of eutrophication for many years in Taihu Lake (Shao et al., 2011; Song et al., 2012; Zeng et al., 2009), P is no longer the only limiting factor of eutrophication eventually. In addition, the genera *Thiobacillus* (phylum *Proteobacteria*) and *Nitrospira* (phylum *Nitrospirae*) were relatively abundant in Gonghu Bay of Taihu Lake, and they were positively correlated with TN concentration (Figure 6a). N forms were affected by *Nitrospirae*, and NO_3_‐N concentration greatly changed in Gonghu Bay of Taihu Lake. Furthermore, *Thiobacillus* can directly participate in NO_3_‐N metabolism, thereby influencing TN concentration (Bruckner et al., 2013; Li, Zhao, & Wang, 2011). Therefore, *Nitrospirae* and *Thiobacillus* are possibly among the main reasons for the change in limiting factors in Gonghu Bay of Taihu Lake. East Dongting Lake is a moderately eutrophic lake, and its eutrophic conditions have recently worsened because of anthropogenic activities and agricultural pollution around its basin. Large amounts of nitrogen have entered into the lake, and nitrogen deposition resulted in relatively high N content in sediments and abundant bacterial communities, such as *Nitrospirae*.

Correlation analysis between genera and physiochemical parameters in sediments showed that the genus *Desulfococcus* (family *Desulfobacteraceae*) was significantly positively correlated with TN and TP concentrations in sediments obtained from East Dongting Lake. In East Dongting Lake, OM content was relatively high, and the transformation of nutrients from inorganic to organic forms occurs frequently, resulting in the association between *Desulfobacteraceae* and organic N or P. Moreover, Figure 6b shows that OM content is positively correlated with the genus *Desulfococcus* in the sediments obtained from East Dongting Lake, and a study has estimated that sulfate reduction can account for as much as 50% of OM degradation (Jorgensen, 1982). Therefore, the high OM content in sediments obtained from East Dongting Lake can ensure the activity of sulfate reducers (Pallud & Van Cappellen, 2006).

In summary, obvious differences in bacterial communities in the three lakes with different trophic statuses were observed, and the bacterial communities were significantly correlated with the trophic status of the lakes. This study comprehensively compared the three lakes with different trophic statuses (moderately eutrophic, lightly eutrophic, and moderately trophic lake) using high‐throughput sequencing method. *Proteobacteria* was the most abundant phylum in these lakes. The sediments from moderately eutrophic lake (Gonghu Bay of Taihu Lake) were enriched with *Chloroflexi* and *Nitrospirae*. The sediment from lightly eutrophic region (East Dongting Lake) showed a low diversity and was enriched with *Alphaproteobacteria*,* Gammaproteobacteria*, and *Bacteroidetes*. The sediments from Huangda Lake, which is a moderately trophic lake, displayed a high abundance of *Acidobacteria* and *Deltaproteobacteria* because of the low pH in sediments in the lake. Temperature could influence the sediment bacterial communities in these lakes. Nutrient concentration was the main factor influencing bacterial communities in Gonghu Bay of Taihu Lake. In East Donging Lake, the organic matter contents could affect the bacterial communities in the sediments, and the bacterial communities in the sediment obtained from Huangda Lake were associated with TN concentration.

## CONFLICT OF INTEREST

None declared.
